# Incorporation of Ln-Doped LaPO_4_ Nanocrystals as Luminescent Markers in Silica Nanoparticles

**DOI:** 10.1186/s11671-016-1465-y

**Published:** 2016-05-21

**Authors:** Jacobine J. H. A. van Hest, Gerhard A. Blab, Hans C. Gerritsen, Celso de Mello Donega, Andries Meijerink

**Affiliations:** Condensed Matter and Interfaces, Debye Institute for Nanomaterials Science, Utrecht University, Princetonplein 5, 3584 CC Utrecht, The Netherlands; Molecular Biophysics, Utrecht University, Princetonplein 5, 3584 CC Utrecht, The Netherlands

**Keywords:** LaPO_4_, Lanthanide, Silica, Nanocrystals, Luminescence

## Abstract

Lanthanide ions are promising for the labeling of silica nanoparticles with a specific luminescent fingerprint due to their sharp line emission at characteristic wavelengths. With the increasing use of silica nanoparticles in consumer products, it is important to label silica nanoparticles in order to trace the biodistribution, both in the environment and living organisms.

In this work, we synthesized LaPO_4_ nanocrystals (NCs) with sizes ranging from 4 to 8 nm doped with europium or cerium and terbium. After silica growth using an inverse micelle method, monodisperse silica spheres were obtained with a single LaPO_4_ NC in the center. We demonstrate that the size of the silica spheres can be tuned in the 25–55 nm range by addition of small volumes of methanol during the silica growth reaction. Both the LaPO_4_ core and silica nanocrystal showed sharp line emission characteristic for europium and terbium providing unique optical labels in silica nanoparticles of variable sizes.

**Electronic supplementary material:**

The online version of this article (doi:10.1186/s11671-016-1465-y) contains supplementary material, which is available to authorized users.

## Background

Nanoparticles find application in an increasing number of consumer products. For example, silica nanoparticles are among the most widely applied nanoparticles. Silica particles of 5–500 nm are used as food additive [1, 2], particles of 50–500 nm in rubber [3, 4], ~25 nm particles as paper additive [5], and 10–250 nm particles in cement [6–8]. Concerns about potential risks of nanoparticles have triggered research on environmental and health issues [1, 9–12]. More research is required to provide insight in the distribution and toxicity of nanoparticles to aid the development of safe-by-design guidelines for manufacturers [13, 14]. For this reason, model systems are needed to make it possible to monitor and map the distribution of nanoparticles. A promising method to trace the nanoparticles is incorporating a luminescent label into nanoparticles. Various luminescent labelled silica systems have been synthesized in the past decades.

Two main approaches have been used to coat luminescent nanoparticles with silica, namely the Stöber method [15, 16] and the reverse micelle method [17–19]. The former method is used for the silica coating of nanoparticles (e.g., 10–200 nm gold and silver particles [20]) in polar media, while the latter method is mainly used for smaller nanoparticles (e.g., 3–10 nm quantum dots [17]) which are suspended in apolar media.

One of the luminescent labels used in silica in the past are quantum dots (QDs) [18]. However, the porous character of the silica limits the stability of the QDs. The surrounding medium can be in contact with the quantum dot which can result in quenching of the luminescence [18]. In addition, the intensity of QD emission decreases after exposure to high temperatures [21]. A second important disadvantage of silica-coated QDs as luminescent labels is the width of the emission bands. Albeit narrower than emission from dye molecules, the 30–40 nm band width limits the number to a few unique luminescent labels that can be created for the QD-labeled silica nanoparticles.

Lanthanide ions incorporated into a nanocrystalline host are a promising alternative since the lanthanide luminescence is known to be unaffected by the surrounding medium, and the chemical and temperature stability of Ln-doped nanocrystals is high [22–24]. In addition, the sharp and characteristic emission lines of lanthanide ions can be used to create a large number of unique luminescent labels by changing or combining the lanthanide ions inside the nanocrystal host lattice [25, 26]. For these reasons, lanthanide-doped insulator nanocrystals are promising as luminescent labels for silica. Here, we will investigate the synthesis and optical properties of silica nanoparticles with a luminescent core of LaPO_4_ doped with luminescent lanthanide ions. Previously, silica coating has been realized for Ln nanostructures with varying size, shape, and type of lanthanide-doped hosts, mostly with the aim to provide a protective silica coating. Examples of these systems are clustered LaPO_4_:Tb^3+^ rods of 10 × 95 nm coated with a 15–20 nm silica layer using the Stöber method [27], CePO_4_:Ln^3+^ nanoleaves of 20 × 75 nm incorporated into silica sols [28], LaF_3_ nanocrystals of 5 nm coated with a silica shell of 17 nm [29], and YVO_4_:Eu^3+^ nanoparticles and LnPO_4_:Ce^3+^/LaPO_4_ core-shell nanoparticles [30, 31] with a thin (5 nm) silica shell. These silica-coated nanoparticles have a thin silica shell and are not representative of silica nanoparticles used in consumer products. It is the aim of this paper to synthesize silica nanoparticles with a unique and efficiently luminescing core that can serve to trace silica nanoparticles of sizes and shapes that reflect those of commercially applied silica nanoparticles.

We present a new method to incorporate LaPO_4_ nanocrystals doped with either europium (Eu^3+^) or cerium and terbium (Ce^3+^ and Tb^3+^) into monodisperse silica spheres using the reverse micelle method. The size of the LaPO_4_ core particles can be varied from 4 to 8 nm by changing the lanthanide precursor to ligand ratio. The size of the silica spheres can be varied between 25 and 55 nm. These sizes are in the same range as for silica nanoparticles that are commonly used in consumer products which make these luminescent-labeled silica nanoparticles relevant for studies of the environmental distribution of silica nanoparticles. In addition, this system may be suitable to perform measurements on luminescent-labeled silica on a single nanoparticle level because of the large number of luminescent lanthanide ions incorporated in a single nanocrystal. This will be useful in in vivo studies of the distribution of silica in living organisms. Finally, these silica particles can also be used as multifunctional biolabels by combining the luminescent core with additional functionalities (e.g., MRI contrast agent, drug delivery) incorporated in the silica shell.

## Methods

### Chemicals

The chemicals used in the various synthesis procedures are LaCl_3_.6H_2_O (Strem chemicals, 99.9 %), EuCl_3_.6H_2_O (Fisher Scientific, 99.9 %), CeCl_3_ (Aldrich, 99.99 %), TbCl_3_.6H_2_O (Aldrich, 99.9 %), tributyl phosphate (Fluka Analytical, ≥99 %), diphenyl ether (Sigma-Aldrich, 99 %), tributylamine (Sigma-Aldrich, ≥99 %), tridodecylamine (Aldrich, 85 %), phosphoric acid (Aldrich, ≥99.9 %), dihexyl ether (Aldrich, 97 %), dodecylamine (Acros Organics, 98 %), decylamine (Aldrich, 95 %), poly(5)oxyethylene-4-nonylphenyl-ether (Igepal Co 520, Sigma-Aldrich), tetraethyl orthosilicate (TEOS, Sigma-Aldrich, 99 %), and ammonia 28 % in water stored at 7 °C (Sigma-Aldrich, 99.9 %) were used as received. The solvents used are methanol (Sigma-Aldrich, 99.8 %), cyclohexane (Sigma-Aldrich, anhydrous, 99.5 %), ethanol (Alfa Aeasar, 96 %), and toluene (Sigma-Aldrich, anhydrous, 99.8 %) and were used as received.

### Synthesis of LaPO_4_ Nanocrystals

LaPO_4_ nanocrystals doped with lanthanide ions were synthesized using a method pioneered in the group of Haase [32]. A clear solution of 10 mmol lanthanide chlorides (La, Eu, or Ce and Tb) in 10 mL methanol was mixed with 40 mmol tributyl phosphate. Subsequently, methanol was removed under vacuum at room temperature in a Schlenk-line. Next, 30 mL of diphenyl ether was added and water released by the hydrated salts was removed under vacuum at 105 °C. The system was purged with nitrogen in a Schlenk-line and the temperature was allowed to drop. At temperatures below 50 °C, 2.5 to 40 mmol tributylamine was added, followed by 7 mL of a 2 M solution of phosphoric acid in dihexyl ether. The reaction mixture was kept overnight (~16 h) under nitrogen at 200 °C to allow for particle growth to the final size (4–8 nm) and annealing of the nanocrystals. After cooling, the nanocrystals were precipitated from the reaction mixture by addition of toluene, washed with methanol and toluene, and dried under vacuum. The nanocrystals could be redispersed in polar media.

### Ligand Exchange

We can vary the medium in which the nanocrystals can be suspended from polar to apolar by changing the ligand attached to the surface of the nanocrystal. A ligand exchange reaction was performed in order to change the short tributylamine ligand with the longer dodecylamine ligand. Recapping of the nanocrystals was performed by adding the dry nanocrystals to dodecylamine heated at 200 °C under nitrogen atmosphere. After 10 min, the heating was stopped and the nanocrystals were precipitated from the reaction mixture by adding methanol followed by centrifugation. The nanocrystals were washed several times with toluene and methanol and dried under vacuum. The nanocrystals could be dispersed in apolar solvents after this recapping procedure.

### Silica Coating of LaPO_4_ Nanocrystals

Silica shells were grown around the LaPO_4_ nanocrystals using the inverse micelle method described by Koole et al. [18]. First, 1.3 mL of Igepal Co 520 (NP-5) was dispersed in 10 mL cyclohexane and stirred at 850 rpm for 15 min. Next, 1–2 nmol tributylamine-capped LaPO_4_ nanocrystals in 100 μL methanol or 1–2 nmol dodecylamine-capped LaPO_4_ nanocrystals in 1 mL toluene were injected. In a number of syntheses, 50 to 150 μL methanol was added directly after the addition of the nanocrystals to vary the silica particle size. Subsequently, 80 μL tetraethyl orthosilicate (TEOS) and 150 μL ammonia were added. The reaction mixture was stirred at 850 rpm for 15 min between every addition and for 1 min after the last addition and stored in a dark room for 1 day. The silica-coated LaPO_4_ nanocrystals were isolated from the reaction mixture by addition of 3 mL ethanol and centrifugation at 3000 rpm for 10 min. The sediment was redispersed in 10 mL ethanol and centrifuged at 3000 rpm for 20 min. This last step was repeated but centrifuging for 40 min after which the silica-coated LaPO_4_ nanocrystals were redispersed in 10 mL ethanol.

### Characterization

The purified NCs and silica samples were characterized with transmission electron microscopy (TEM). Samples for analysis were obtained by dissolving 0.5 mg of nanocrystals in 3 mL ethanol and dropcasting the NCs solutions on coated copper TEM grids. The TEM images were obtained with a Tecnai microscope operating at 100 kV equipped with a tungsten filament. Images were recorded with a SIS CCD camera Megaview II in iTEM software.

X-ray diffraction patterns of powder samples were recorded with a PW1729 Philips diffractometer equipped with a Cu Kα X-ray source (*λ* = 1.5418 Å). Reference diffractograms were taken from the International Center of Diffraction Data (ICDD).

### Luminescence Spectroscopy

Photoluminescence measurements were performed using an Edinburgh Instruments FLS920 fluorescence spectrometer. Emission spectra were recorded using a 450 W Xe lamp as excitation source and a Hamamatsu R928 PMT detector. Luminescence decay curves were recorded for pulsed excitation with an optical parametric oscillator (OPO) system (Opotek HE 355 II) pumped by the third harmonic of a Nd:YAG laser. The OPO was set at *λ*_exc_ = 487 nm to excite in the Tb^3+^^7^F_6_ → ^5^D_4_*f-f* transition (repetition rate 10 Hz, pulse width 10 ns) for the LaPO_4_:Ce^3+^,Tb^3+^ nanocrystals. The LaPO_4_:Eu^3+^ nanocrystals were measured with the OPO at *λ*_exc_ = 465 nm to excite in the Eu^3+^^7^F_0_ → ^5^D_2_*f-f* transition. The decay curves were recorded with a Hamamatsu R928 PMT detector using the multichannel scaling (MCS) option integrated in the FLS 920 fluorescence spectrometer.

### Dynamic Light Scattering

The sizes of the micelles formed during silica coating were characterized with dynamic light scattering (DLS). Details can be found in Additional file 1. Four different samples were prepared to analyze the size of the micelles under varying reaction conditions; NP-5 in cyclohexane solutions, ammonia emulsions, and two methanol/ammonia emulsions with different concentrations of methanol. The 306 mM NP-5 in cyclohexane solutions were obtained by dissolving 1.35 g NP-5 in 10 mL cyclohexane under stirring at 850 rpm for 15 min. Ammonia emulsions were prepared by adding 150 μL ammonia (28 wt.% in water) to the NP-5 solutions. The methanol/ammonia solutions were obtained by adding methanol, either 50 or 150 μL, to the NP-5 mixtures, obtaining concentrations of 121 and 359 mM, respectively. After 15 min of stirring at 850 rpm, 150 μL ammonia (28 wt.% in water) was added. All samples were filtered with a Millipore Millex-FG 0.20 μm filter.

## Results and Discussion

### LaPO_4_ Cores

**Fig. 1:**
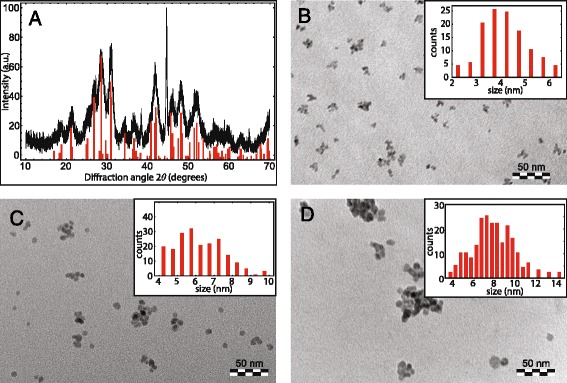
**a** XRD of LaPO4 nanocrystals shown in **c**. The *red bars* indicate the positions and relative intensities of the XRD pattern of bulk LaPO_4_ [PDF 00-032-0493]. **b**–**d** TEM images of LaPO_4_:Eu^3+^ nanocrystals synthesized with different amounts of tributylamine capping ligands. **b** LaPO_4_ nanocrystals of 4.2 ± 0.9 nm for a LnCl3 to ligand ratio of 1:4. **c** LaPO_4_ nanocrystals of 6.1 ± 1.2 nm for a LnCl3 to ligand ratio of 2:1. **d** LaPO_4_ nanocrystals of 7.9 ± 1.8 nm for a 4:1 ratio

The size of both the silica nanoparticles and the luminescent core are important parameters. In this section, we report results on the variation of the LaPO_4_ core diameter. In a larger core, the number of luminescent lanthanide ions that can be incorporated is higher, which can be important to achieve single nanocrystal luminescence. The LaPO_4_ nanocrystal size can be tuned by varying the ratio between NC precursors (Ln and phosphate) and the ligand. Hickmann et al. [32] have observed an increase in particle sizes from 4 to 10 nm by changing the lanthanide precursor to ligand ratio from 1:3 to 1:1. In Fig. 1, X-ray diffraction (XRD) patterns and transmission electron microscope (TEM) images are shown for LaPO_4_ NCs synthesized with precursor ratios varying from 4:1 to 1:4 (details can be found in Additional file 1). Figure 1a shows a XRD pattern of particles shown in Fig. 1c. A monazite LaPO_4_ reference diffractogram is included in the same figure. The X-ray diffractogram shows diffraction peaks that are consistent with the monazite crystal structure for LaPO_4_. The presence of broad diffraction peaks reflects the formation of nanoparticles with sizes in the nanometer range. A more precise determination of the size can be obtained by analyzing the TEM images. In Fig. 1b–d TEM images are shown for LaPO_4_ NC synthesized with different precursor-ligand ratios. For the higher ligand concentrations (Fig. 1 b, c), smaller NCs are formed, with diameters ranging from 4 to 6 nm. The variation of the NC size was not always reproducible. The general trend of the present experiments confirm the earlier findings by Hickmann et al. that a lower ligand concentration results in larger LaPO_4_ NCs. Similar observations are generally made (also for II–VI QDs [33–35]) and are explained by the faster growth of NCs that are less well capped/protected by ligands.

**Fig. 2:**
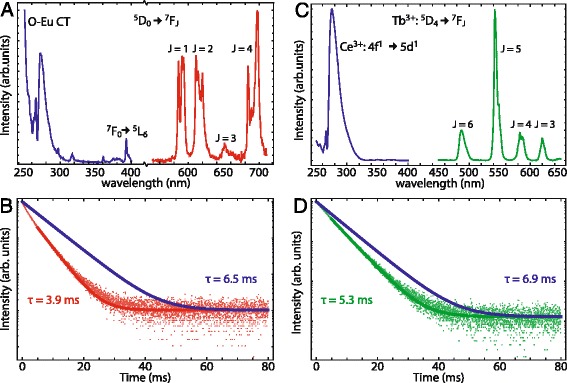
**a**: Emission spectrum (*red line*, *λ*_exc_ = 250 nm) and excitation spectrum (*blue line*, *λ*_em_ = 611 nm) of 4 nm LaPO_4_:Eu^3+^ NCs in ethanol. **b** Decay curve (*red line*) of 611 nm Eu^3+^ emission for 4 nm LaPO_4_:Eu^3+^ NCs in ethanol excited at 465 nm, *τ* = 3.9 ms, and the radiative decay curve with QY = 1 (*blue line*), *τ* = 6.5 ms. **c** Emission spectrum (*green line*, *λ*_exc_ = 280 nm) and excitation spectrum (*blue line*, *λ*_em_ = 543 nm) of 4 nm LaPO_4_:Ce^3+^, Tb^3+^ in ethanol. **d** Decay curve of 543 nm Tb^3+^ emission for 4 nm LaPO_4_:Ce^3+^(1 %),Tb^3+^(5 %) NCs in ethanol excited at 487, *τ* = 5.3 ms, and the purely radiative decay curve (*blue line*), *τ* = 6.9 ms. See text for details concerning the calculation of the purely radiative decay curves

To investigate the optical properties of the doped LaPO_4_ NCs, luminescence spectra and luminescence decay curves were recorded for the various types of NCs (different sizes and different types of dopants). Figure 2a shows the emission spectrum (red line) of LaPO_4_:Eu^3+^(5 %) nanoparticles of 4 nm. Sharp emission lines are observed around 590, 610, and 700 nm. The sharp emission lines are typical of Eu^3+^ emission corresponding to intraconfigurational 4f^6^ transitions. The strongest emission lines can be assigned to the ^5^D_0_–^7^F_1_ (590 nm), ^5^D_0_–^7^F_2_ (610 nm), and ^5^D_0_–^7^F_4_ (700 nm) transitions. The relative intensities of these three emissions are similar. This is in good agreement with reports in the literature for Eu^3+^-doped LaPO_4_ NCs [36] and bulk [37]. When Eu^3+^ ions are incorporated on a site that lacks inversion symmetry (which is the case for the La^3+^ site in LaPO_4_), usually the ^5^D_0_ → ^7^F_2_ emission dominates. However, for Eu^3+^ in LaPO_4_, the mixing of opposite parity states into the 4f^6^ states by the odd parity crystal field components, the mechanism responsible for breaking the parity selection rule for electric dipole transitions is limited, resulting in approximately equal intensities of the ^5^D_0_ → ^7^F_1_ magnetic dipole transition and ^5^D_0_ → ^7^F_2_ electric dipole transition [37, 38]. The blue line in Fig. 2a shows the excitation spectrum of the Eu^3+^ emission. The onset of the excitation at 300 nm is followed by increasing absorption towards shorter wavelengths. The strong UV excitation band originates from the oxygen to europium charge transfer (CT) band [37, 39]. The dip in the excitation band around 270 nm is ascribed to competitive absorption by organic molecules present in the solution, possibly some residual diphenyl ether that was used as solvent and has an absorption maximum around 270 nm or molecules with multiple unsaturated bonds that are formed during the reaction at 200 °C and which typically absorb at these energies [40]. Next to the parity allowed CT transition with high intensity, sharp, and weak excitation peaks are observed between 300 and 400 nm and can be assigned to the intraconfigurational transitions to higher energy 4f^6^ levels.

Figure 2b shows the luminescence decay curve of the Eu^3+^ emission in 4 nm LaPO_4_:Eu^3+^ 5 % NCs, recorded for 611 nm emission. After an initial non-exponential decay, the tail of the decay curve is closer to single exponential. A mono-exponential fit to these data points gives a decay time of 3.9 ms. The initial fast decay can be explained by an additional contribution of non-radiative decay pathways caused by multi-phonon relaxation through nearby N–H and C–H vibrations (phonons) of ligand molecules with energies of ~3400 and 3000 cm^−1^, respectively. The energy gap between the emitting ^5^D_0_ level and the next lower ^7^F_6_ level is about 12,000 cm^−1^ and can be bridged by four N–H or C–H vibrations. Multi-phonon relaxation and radiative decay rates are similar for energy gaps that can be bridged by five phonons [24]. Multi-phonon relaxation becomes faster if the gap can be bridged by a smaller number of phonons and when the distance to the vibrational oscillations decreases. Especially Eu^3+^ ions close to the surface will show faster decay due to multi-phonon relaxation caused by coupling with the nearby C–H and N–H vibrations. The fast initial decay and influence of the differences in coordination for ions at the surface on the luminescence and decay behavior will be discussed in a next paper.

The radiative decay time of emitters in NCs depends strongly on the refractive index *n* of the medium surrounding the NC. Calculating the radiative decay time and comparison with the experimentally observed decay curve allows an estimate of the upper limit for the quantum yield (QY) of luminescent NCs [41]. The radiative decay rate can be determined using formula 5 in ref [41]:

1 $$ {\varGamma}_r(n) = {\varGamma}_0n{\left(\frac{3{n}^2}{2{n}^2+{n}_{\mathrm{NC}}^2}\right)}^2 $$

The calculated *Γ*_r_(*n*) was determined to be 0.16 ms^−1^ (6.5 ms), using *Γ*_0_ = 0.31 ms^−1^ (*τ* = 3.18 ms for bulk LaPO_4_:Eu^3+^(2 %) [42]), *n*_NC_ = 1.79 [43], and *n* = 1.361 (ethanol). The value of 6.5 ms is longer than the experimentally observed decay time of 3.9 ms in the tail of the decay curve. Consequently, the influence of the non-radiative decay is still present in the tail of the decay curve. An estimate for the upper limit of the QY can be obtained by dividing the area under the measured decay curve by the area under the theoretically determined decay curve obtained by Eq. 1 [41]. This procedure gives a quantum efficiency of 0.48.

The emission spectrum of LaPO_4_:Ce^3+^(1 %),Tb^3+^(5 %) NCs is shown by the green line in Fig. 2c. Emission lines are observed around 490, 540, 585, and 620 nm. The sharp emission lines are attributed to ^5^D_4_ to ^7^F_J_ transitions and agree with typical Tb^3+^ emission lines reported in the literature [44]. The green ^5^D_4_–^7^F_5_ emission around 545 nm dominates as is usually observed for Tb^3+^. The blue line in Fig. 2c shows the excitation spectrum of terbium emission. An excitation band starting at 325 nm is observed and assigned to the parity-allowed 4f → 5d transition of Ce^3+^. The band abruptly drops in intensity at 270 nm, which is similar to the wavelengths where a drop in the excitation spectrum for the CT band of Eu^3+^ was observed. The drop in intensity is again explained by competing absorption of UV radiation in this wavelength region by organic molecules with a conjugated π-system (multiple alternating double bonds). The observation of the Ce^3+^ excitation bands while monitoring Tb^3+^ emission provides evidence that there is energy transfer from Ce^3+^ to Tb^3+^ [44].

The luminescence decay curve of the ^5^D_4_ emission from Tb^3+^ in 4 nm LaPO_4_ NCs doped with Ce^3+^ and Tb^3+^ recorded at 543 nm is shown in Fig. 2d. A mono-exponential fit for the tail of the decay curve, starting 5 ms after the excitation pulse, yields a decay time *τ* of 5.3 ms. Just as for Eu^3+^, the initial part of the luminescence decay curve is non-exponential but the deviation from exponential decay is less than for Eu^3+^. This originates from the difference in energy between the ^5^D_0_–^7^F_6_ energy gap for Eu^3+^ (~12000 cm^−1^) and the ^5^D_4_–^7^F_0_ energy gap for Tb^3+^ (~14500 cm^−1^). The former can be bridged by four phonons (*vide supra*), while the latter requires five phonons. As a result, the non-radiative decay process is more pronounced in Eu^3+^. An upper limit for the quantum yield of 0.74 was obtained for LaPO_4_:Ce^3+^(1 %),Tb^3+^(5 %) NCs using the same procedure as described above, based on Eq. (5) of ref. [41]. The radiative decay rate, *Γ*_*r*_(*n*), was determined to be 0.145 ms^−1^ when using the constants *Γ*_0_ = 0.29 ms^−1^ (*τ* = 3.4 ms for bulk LaPO_4_:Tb^3+^(1 %) [41]), *n*_NC_ = 1.79, *n* = 1.363 (ethanol).

### Silica-Coated LaPO_4_ NCs

In order to grow a silica nanoparticle around the luminescent LaPO_4_:Ln^3+^ NC cores, an inverse micelle method was used with variations in the reaction conditions. Reaction conditions were varied aiming at growing monodisperse silica NPs of different sizes in the 10–100 nm size range—that is most relevant in view of the current commercial applications of silica nanoparticles. First, the LaPO_4_:Ln^3+^ NCs were suspended in apolar media for the silica-coating reaction according to the procedure described by Koole et al. [18]. In the work of Koole et al., quantum dots capped with long apolar ligands were used for the silica coating and suspended in an apolar medium that was injected in the inverse micelle solution. In order to follow this as accurately as possible, the 4 nm LaPO_4_:Eu^3+^ and LaPO_4_:Ce^3+^,Tb^3+^ NCs coated with tributylamine were subjected to a ligand exchange reaction with dodecylamine. These NCs could be dispersed into apolar media and were dispersed in toluene for the silica-coating reactions prior to injection in the inverse micelle solution.

**Fig. 3:**
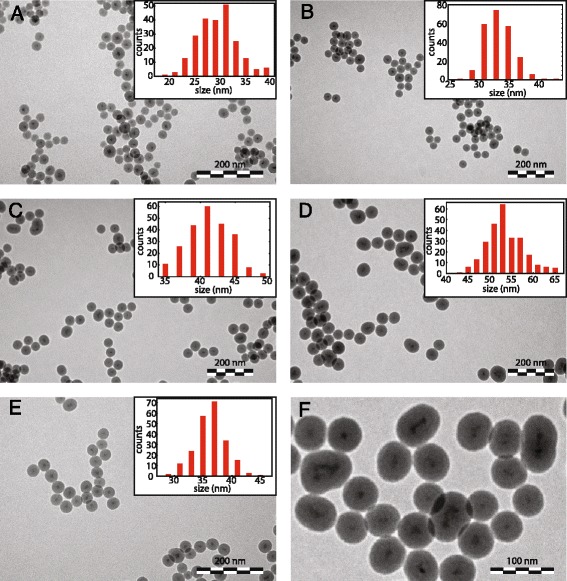
TEM images of silica nanoparticles with a luminescent LaPO_4_ core. The medium in which the LaPO_4_ nanocrystals were dispersed prior to silica growth and the amount of methanol added during the reaction was varied resulting in different SiO_2_ particle sizes. **a** Particles of 28.3 ± 3.8 nm are obtained for DDA-capped LaPO_4_ particles suspended in toluene, no methanol added. **b** Particles of 33.4 ± 2.3 nm, similar to **a** with 50 μL methanol added. **c** Particles of 41.2 ± 3.1 nm, similar to **a** with 100 μL methanol added. **d** Particles of 53.5 ± 4.2 nm, similar to **a** with 150 μL methanol added. **e** Particles of 36.4 ± 2.8 nm are obtained for tributylamine-capped LaPO_4_ particles suspended in 100 μL methanol. **f** Zoom of silica particles shown in **d**

The silica growth reaction was stopped after 1 day, and the final size and shape of the particles were studied with TEM. Figure 3a shows a TEM image of the LaPO_4_ crystals after silica coating. Spherical silica particles with a size of 28.3 ± 3.8 nm are obtained. The LaPO_4_ NCs are located in the center of the sphere and many silica spheres contain only a single LaPO_4_ NC. However, silica spheres with no or multiple LaPO_4_ NCs are observed as well. These empty silica particles have a smaller size than particles containing one or multiple LaPO_4_ NCs.

For investigating biodistribution of silica particles of different sizes, it is desired to be able to tune the size of the silica particles. However, the final silica particle size can only be tuned within a small size range with the method described above. To vary the size of the silica NPs, we added a varying amount of methanol directly after the addition of the LaPO_4_ NCs to the silica reaction mixture to study the influence on the size of the silica spheres. The added volume of methanol was increased from 50–150 μL in steps of 50 μL. More information about experimental details can be found in Additional file 1. Figure 3b–d shows the NCs after silica coating with increasing methanol concentration going from 3b to 3d. The 4 nm LaPO_4_ NCs are incorporated in the middle of the silica spheres, and the size of the silica spheres increases from 33.4 ± 2.3 nm to 41.2 ± 3.1 to 53.5 ± 4.2 nm with increasing methanol concentration. This demonstrates that the presence of methanol during the silica reaction increases the silica growth around the LaPO_4_ NCs.

The variation in size is larger for silica particles synthesized in the presence of a higher volume of methanol. The size of the silica particles is dependent on the number of LaPO_4_ NCs incorporated, where larger silica particles are formed as more LaPO_4_ NCs are incorporated. A series of experiments under slightly varying reaction conditions (e.g., source of ammonia) consistently reproduced the effect of the addition of methanol on particle size. Depending on other reaction parameters also larger silica NPs, up to 80 nm, were obtained, see Figure S1 in Additional file 1. In all cases, an addition of small volumes (50–200 μL) of methanol showed similar increases of silica particle sizes.

It is not trivial to explain the role of methanol on the final particle size. In the literature, it has been shown that the addition of alcohol to microemulsions results in coalescence of micelles [45]. In this mechanism, the micelles should enlarge upon addition of alcohol. This is indeed observed for addition of methanol or ethanol to AOT/water/*n*-decane systems [46]. In our system, another surfactant and oil phase is used and it is not evident that the two systems will behave in the same way. Dynamic light scattering (DLS) measurements can provide information about the size of micelles and is used to study the size of micelles under varying reaction conditions. DLS measurements performed on 306 mM NP-5 in cyclohexane showed the presence of particles with a mean hydrodynamic diameter of 3.5 ± 1.3 nm. The size of the particles increases to 6.3 ± 2.2 nm upon adding ammonia solution (28 wt.% in water) to the system. The increase in size indicates that the ammonia solution is incorporated into the micelles, forming small emulsion droplets.

To investigate the influence of methanol on the micelle system, two different concentrations methanol were added to the mixture of NP-5, ammonia solution and cyclohexane and investigated with DLS (Additional file 1). The hydrodynamic diameter of the emulsion droplets shifted to 7.8 ± 4.0 nm for systems containing 121 mM methanol. In addition, a second size at approximately 1700 nm was observed. However, only a few large structures are present as can be seen from the number distribution. DLS measurements performed on a system containing a higher concentration methanol, 359 mM, did not indicate a significant increase in droplet size. These results indicate that the micelles remain intact and most likely increase significantly in size after the addition of methanol to the system, probably due to the partly incorporation of methanol. In addition, a small number of structures with a large diameter are formed. Table S1 in Additional file 1 lists the data in more detail.

A possible explanation for the formation of larger silica spheres in the presence of methanol is the formation of less silica nuclei in the beginning of the reaction due to the formation of hydrogen bonds between methanol and water. In the literature, it has been shown that an increase in the water concentration leads to the formation of smaller silica particles [47]. This observation is explained by the faster hydrolysis rate of the silicon source, tetraethyl orthosilicate (TEOS), in the presence of more free water. The fast hydrolysis of TEOS leads to the formation of a larger number of stable silica nuclei. Since all nuclei grow at the same rate, more but smaller silica particles are formed at the end of the reaction, when all TEOS has reacted. It is therefore expected that the presence of less free water leads to the formation of fewer but larger silica particles. Possibly, the amount of free water is reduced by adding methanol to the reaction mixture, since the alcohol can form hydrogen bonds with water. However, it is beyond the scope of this paper to unravel in detail the mechanism responsible for the increase in silica particle size upon addition of small volumes of methanol. The present study clearly demonstrates that this new method can be successfully applied to tune the size of silica NPs over a relevant size range.

Another variation in reaction parameters was made to grow silica around the LaPO_4_ NCs starting from a suspension of LaPO_4_ nanocrystals in polar media. For this coating reaction, the as-synthesized LaPO_4_ NCs coated with tributylamine were suspended in 100 μL methanol and injected into the inverse micelle solution. The reaction was stopped after 1 day and the final particles are shown in Fig. 3e. Spherical particles with a diameter of 36.4 ± 2.8 nm are observed with one or multiple LaPO_4_ NCs in the middle. The size of the NCs is comparable to the size of the silica NCs shown in Fig. 3c. The total amount of methanol is the same in both reaction mixtures, but in the latter, the LaPO_4_ NCs were suspended in apolar medium and later methanol was added to the reaction mixture. This indicates similar silica growth mechanisms for both silica-coating reactions. Again, larger silica NCs are formed in reaction mixtures with a higher concentration methanol. The size of the silica spheres can be tuned from 28 to 54 nm by adjusting the amount of methanol between 0 and 150 μL.

**Fig. 4:**
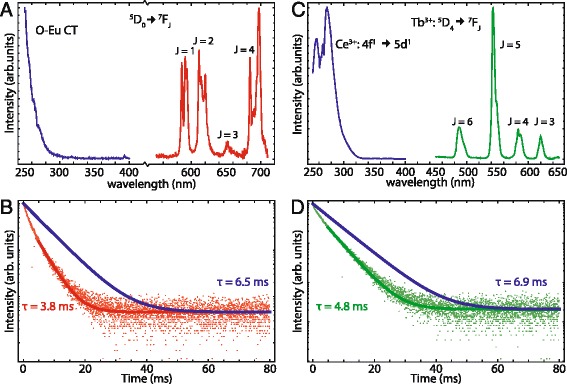
**a** Emission spectrum (*red line*, *λ*_exc_ = 250 nm) and excitation spectrum (*blue line*, *λ*_em_ = 611 nm) of LaPO_4_:Eu^3+^ nanocrystals incorporated into silica spheres dispersed in ethanol. **b** Decay curve of 611 nm Eu^3+^ emission for 4 nm LaPO_4_:Eu^3+^ NCs incorporated in 28 nm silica spheres dispersed in ethanol excited at 465 nm, *τ* = 3.8 ms, and the radiative decay curve with QY = 1 (*blue line*), *τ* = 6.5 ms. **c** Emission spectrum (*green line*, *λ*_exc_ = 280 nm) and excitation spectrum (*blue line*, *λ*_em_ = 543 nm) of LaPO_4_:Ce^3+,^Tb^3+^ NCs incorporated in silica spheres dispersed in ethanol. **d** Decay curve of LaPO_4_:Ce^3+,^Tb^3+^ NCs incorporated in silica spheres dispersed in ethanol excited at 487 nm, *τ* = 4.8 ms, and the radiative decay curve with QY = 1 (*blue line*), *τ* = 6.9 ms. See text for details concerning the calculation of the purely radiative decay curves

For application of the silica NPs in studying biodistribution, it is crucial that the luminescence properties are retained after silica growth. Luminescence spectra were measured for silica NPs with LaPO_4_:Eu^3+^ and LaPO_4_:Ce^3+^,Tb^3+^ nanocrystal cores. Luminescence spectra are shown in Fig. 4.

The Eu^3+^ emission spectrum (red line in Fig. 4a) is identical to that in Fig. 2a which shows that the europium emission is not affected after silica growth. In addition, the ratio between the emission intensities of the ^5^D_0_ to ^7^F_1_, ^7^F_2_, and ^7^F_4_ transitions is the same as for the LaPO_4_:Eu^3+^ cores. This is expected since the local surrounding of the europium ions is determined by the local coordination in the LaPO_4_ crystal structure and is not changed after silica growth. The excitation spectrum, blue line in Fig. 4a, shows a broad excitation band with an onset around 300 nm, originating from oxygen to europium charge transfer, and a small peak at 393 nm, assigned to the ^7^F_0_ → ^5^L_6_ transition. A single broad CT excitation band is observed in the UV without dips around 270 nm which caused the double band structure in the CT band of Eu^3+^ for the LaPO_4_ cores (Fig. 2a). This is consistent with the removal of the organic ligands as a result of the replacement by silica at the LaPO_4_ nanoparticle surface.

The luminescence decay curve of the Eu^3+^ emission in LaPO_4_:Eu^3+^ 5 % NCs incorporated into 28 nm silica spheres, recorded at 611 nm, is shown in Fig. 4b. A mono-exponential fit for the tail of the decay curve, after 5 ms, yields a decay time *τ* of 3.8 ms. The decay time is similar to the decay time of the LaPO_4_:Eu^3+^ cores (3.9 ms).

The upper limit of the quantum yield of the silica-coated LaPO_4_:Eu^3+^(5 %) was determined using the same method described above to be 0.45. A theoretical decay rate *Γ*_*r*_(*n*) of 0.155 ms^−1^ (6.5 ms) was obtained, using *Γ*_0_ = 0.31 ms^−1^, *n*_NC_ = 1.79, and *n* = 1.361 (ethanol). The quantum yield stayed approximately the same from 0.48 before silica coating to 0.45 after silica coating. Note that the value for *n* is not exact since the presence of the silica layer around the LaPO_4_:Eu^3+^ NC should also be taken into account as the higher refractive index of silica (*n* = 1.45) will affect the local field correction factor. Calculations using a method describing the local field correction factor for a core-shell system [48] show that this hardly influences the QY calculated from the luminescence decay curves for these particles. More details can be found in Additional file 1.

The green line in Fig. 4c shows the emission spectrum of terbium in LaPO_4_ NCs incorporated into silica. The blue line shows the excitation spectrum recorded for 543 nm emission. A broad excitation band with an onset at 300 nm is observed and assigned to the parity-allowed 4f → 5d transition of Ce^3+^. A small dip is observed in the excitation band around 270 nm which can be the result of some residual ligands absorbing in the UV or crystal field splitting of the 5d excited state of Ce^3+^ which has been observed to give structure in the 250–300 nm f–d absorption band of Ce^3+^ in LaPO_4_ [38, 49].

The luminescence decay curve of the 543 nm Tb^3+^ emission in LaPO_4_ NCs doped with 1 % Ce^3+^ and 5 % Tb^3+^ incorporated into 28 nm silica spheres is shown in Fig. 4d. A mono-exponential fit for the tail of the decay curve, after 5 ms, yields a decay time *τ* of 5.0 ms and is plotted through the data points. Again, an upper limit for the quantum yield was determined and a value of 0.55 was obtained. The theoretical decay rate, *Γ*_*r*_(*n*), was determined to be 0.16 ms^−1^ when using the constants *Γ*_0_ = 0.29 ms^−1^ (*τ* = 3.4 ms for bulk LaPO_4_:Tb^3+^(1 %) [41]), *n*_NC_ = 1.79, and *n* = 1.363 (ethanol). The quantum yield has decreased from 0.74 before silica coating to 0.55 after silica coating.

The drop in quantum yield after the coating of the LaPO_4_:Ce^3+^,Tb^3+^ nanoparticles can be caused by several different mechanisms, such as increase in multiphonon relaxation and the presence of more defects at the surface of the LaPO_4_ nanocrystal. The decrease in quantum yield can be explained by an increase in the non-radiative decay rate due to faster multiphonon relaxation after silica coating. The N–H and C–H vibrations of ligands with energies of ~3400 and 3000 cm^−1^, respectively, contribute to multiphonon relaxation before silica coating. After silica coating, the ligands are replaced by silanol groups [50]. The O–H vibrations have energies of ~3500 cm^−1^. As a result, the ^5^D_4_–^7^F_0_ energy gap of Tb^3+^ (~14500 cm^−1^) can be bridged by four phonons in the presence of silica instead of five phonons before silica coating, leading to a faster non-radiative decay rate. In addition, the enhanced non-radiative decay rate after silica coating leads to a lower quantum yield. The drop in quantum yield after silica coating is not observed for europium-doped nanoparticles. This is surprising. To explain this observation, we consider the number of phonons (originating from vibrations of ligands) required to bridge the ^5^D_0_–^7^F_6_ energy gap (~12000 cm^−1^) of europium. This number is four, both for the core nanoparticles (N–H and C–H vibrations of ~3400 and 3000 cm^−1^, respectively) and the silica-coated particles (O–H vibrations of ~3500 cm^−1^). As a result, the non-radiative decay rate of europium ions is not significantly increased by the silica growth. The similar multiphonon relaxation rates also result in similar quantum yields for europium-doped LaPO_4_ core NCs and silica-coated nanoparticles.

Next to vibrations of capping materials, vibrations of solvent molecules can also contribute to multiphonon relaxation. The surface of the LaPO_4_ nanocrystal is less well protected from solvent molecules after silica coating due to the removal of ligands and the porous character of silica. As a result, the OH groups of the solvent molecules (ethanol) can couple more easily with the emission of the lanthanide ions located at the surface of the LaPO_4_ nanocrystal. Again, the ^5^D_4_–^7^F_0_ energy gap of terbium (~14,500 cm^−1^) can be bridged by five phonons (N–H and C–H vibrations of ~3400 and 3000 cm^−1^, respectively) before silica coating, while only four phonons are needed after silica coating (O–H vibrations of ~3500 cm^−1^). The number of phonons required to bridge the ^5^D_0_–^7^F_6_ energy gap (~12,000 cm^−1^) of europium is four, both for the core nanoparticles and the silica-coated particles. As a result, the quantum yield of terbium-doped LaPO_4_ NCs drops after silica coating, while the quantum yield of europium-doped LaPO_4_ NCs remains approximately the same after silica coating, as explained before.

The results presented demonstrate the feasibility of making highly luminescent silica nanoparticles of various sizes showing characteristic Eu^3+^ or Tb^3+^ emission. An almost unlimited variety of unique luminescent labels can be realized by incorporating other luminescent Ln^3+^ ions (e.g., Pr^3+^, Sm^3+^, Dy^3+^, Ho^3+^, Er^3+^, Tm^3+^, or Yb^3+^) in the LaPO_4_ core or combinations of different Ln^3+^ ions. It is also interesting to extend the size range by growing additional layers of silica around the nanoparticles.

## Conclusions

LaPO_4_ nanocrystals doped with either europium or cerium and terbium with sizes varying from 4 to 8 nm were synthesized by adjusting the lanthanide precursor to ligand ratio. The LaPO_4_ NCs showed sharp emission lines characteristic for europium or terbium emission.

In a next step, the LaPO_4_ nanocrystals were incorporated in silica nanoparticles using a reverse micelle method. Monodisperse silica spheres with a single LaPO_4_ NC or multiple LaPO_4_ NCs were obtained. Silica particle sizes could be tuned between 25 and 55 nm by addition of small volumes (0 to 150 μL) of methanol. The luminescence spectra of LaPO_4_:Ln^3+^ cores are not affected by silica growth, and the quantum yield remains high after encapsulation in silica nanoparticles. The sizes of the silica nanoparticles studied here is comparable to those of silica nanoparticles applied in consumer products, and the method presented allows for the synthesis of a variety of uniquely labelled silica nanoparticles for biodistribution studies of silica nanoparticles, even down to single nanocrystal experiments using a combination of fluorescence and electron microscopy.

## Additional file

Additional file 1: Supporting information. (910 KB)
