# Supplementary material for: Incorporation of Ln-Doped LaPO4 Nanocrystals as Luminescent Markers in Silica Nanoparticles
Source: Nanoscale Res Lett. 2016 May 21;11:261. doi: 10.1186/s11671-016-1465-y (PMC4875915; doi:10.1186/s11671-016-1465-y)
Supplement: Additional file 1: — Supporting information. (910 KB) [file 11671_2016_1465_MOESM1_ESM.docx]

**Supporting information**

**Incorporation of Ln-doped LaPO_4_ nanocrystals as luminescent markers in silica nanoparticles**

Jacobine van Hest^1,2^, Gerhard Blab^2^, Hans Gerritsen^2^, Celso de Mello Donega^1^ and Andries Meijerink^1*^

*^1^Condensed Matter and Interfaces, Debye Institute for Nanomaterials Science, Utrecht University, Princetonplein 5, 3584 CC Utrecht, The Netherlands*

^2^ *Molecular Biophysics, Utrecht University, Princetonplein 5, 3584 CC Utrecht, The Netherlands*

***Corresponding Author**

A.Meijerink@uu.nl

**Experimental section**

*Synthesis of LaPO_4_ core particles with different sizes*

LaPO_4_ nanocrystals were synthesized by the method described by Hickmann [1]. A clear solution of 10 mmol lanthanide chlorides (La, Eu, or Ce and Tb) in 10 mL methanol was mixed with 40 mmol tributyl phosphate. Subsequently, methanol was removed under vacuum at room temperature in a Schlenk-line. Next, 30 mL of diphenyl ether was added and water released by the hydrated salts was removed under vacuum at 105°C. The system was purged with nitrogen in a Schlenk-line and the temperature was allowed to drop. The reaction mixture was cooled to below 50°C. Different amounts of tributylamine were added, namely 40, 5 or 2.5 mmol, in order to obtain LaPO_4_ nanocrystals (NCs) with a diameter of 4, 6 or 8 nm, respectively. Directly after the addition of tributylamine, 7 mL of a 2M solution of phosphoric acid in dihexyl ether was injected. The reaction mixture was kept overnight (~16 h) under nitrogen at 200°C to allow for particle growth to the final size (4-8 nm) and annealing of the nanocrystals. After cooling, the nanocrystals were precipitated from the reaction mixture by addition of toluene, washed with methanol and toluene, and dried under vacuum. The nanocrystals could be redispersed in polar media.

*Silica coating of LaPO_4_ nanocrystals*

Silica shells were grown around the LaPO_4_ nanocrystals using the inverse micelle method described by Koole et al. [2]. First, 1.3 mL of Igepal Co 520 (NP-5) was dispersed in 10 mL cyclohexane and stirred at 850 rpm for 15 minutes. Next, 1-2 nmol dodecylamine-capped LaPO_4_ nanocrystals in 1 mL toluene were injected. In a number of syntheses, 50 to 150 µL methanol was added directly after the addition of the nanocrystals to vary the silica particle size. Addition of 0, 50, 100 or 150 μL methanol to the reaction mixture resulted in the formation of silica spheres with sizes of 28, 33, 41 and 53 nm, respectively. Subsequently, 80 μL tetraethyl orthosilicate (TEOS) and 150 μL ammonia were added. The reaction mixture was stirred at 850 rpm for 15 minutes between every addition and for 1 minute after the last addition and stored in a dark room for 1 day. The silica-coated LaPO_4_ nanocrystals were isolated from the reaction mixture by addition of 3 mL ethanol and centrifugation at 3000 rpm for 10 minutes. The sediment was redispersed in 10 mL ethanol and centrifuged at 3000 rpm for 20 minutes. This last step was repeated but centrifuging for 40 minutes after which the silica-coated LaPO_4_ nanocrystals were redispersed in 10 mL ethanol.

*Characterization: Dynamic light scattering*

DLS experiments were performed on a Malvern Zetasizer Nano instrument. Glass cuvettes were used. All measurements were performed in seven runs of at least 15 individual measurements in backscatter mode (scattering angle θ = 173°) at 298 K.

For monodisperse particles in Brownian motion, the intensity correlation function measured is

$$G\left( \tau\right)=1+e^{-2q^{2}D^{trans}\tau}$$

where τ is the correlation time and q is the magnitude of the scattering vector and is described by

$$q=\frac{4\pi n}{\lambda}sin\frac{\theta}{2}$$

where θ is the scattering angle of incident light, λ is the wavelength of light in vacuum and *n* is the refractive index of the solution. A laser operating at 632.8 nm was used and the refractive index of cyclohexane is 1.42. The translational diffusion coefficient, D^trans^, relates to the hydrodynamic diameter, d^h^, following the Stokes-Einstein relation:

$$d^{h}=\frac{kT}{3\pi\eta D^{trans}}$$

where d^h^ is a spherical particle, k is the Boltzmann constant, T the temperature and η the viscosity of the solution. A viscosity of 0.98 mPa.s was used for cyclohexane at 298 K. The reported sizes are the mean hydrodynamic diameters and the polydispersity the peak width obtained from the average of 7 runs.

**Results**

*TEM of silica nanoparticles with LaPO_4_ core*

| 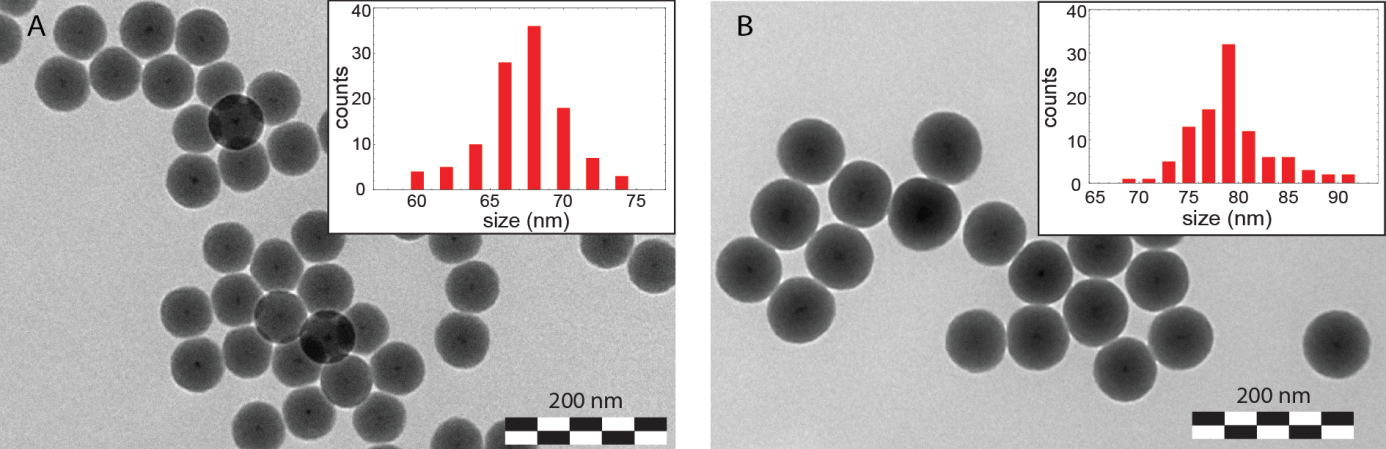 |
| --- |
| Figure S1. TEM images of silica nanoparticles with a luminescent LaPO_4_ core. The number of LaPO_4_ nanocrystals added for silica growth is 0.1 nmol instead of 0.5 nmol for silica particles obtained in Figure 3 of the main text. The amount of methanol added during the reaction was varied resulting in different SiO_2_ particle sizes. A: Particles of 67.4 ± 2.9 nm are obtained for DDA-capped LaPO_4_ particles suspended in cyclohexane, 100 μL methanol added. B: Particles of 79.4 ± 4.6 nm, similar to A with 200 μL methanol added. |

*Dynamic Light scattering*

| Table S1. Mean diameters and polydispersity of various samples obtained from the intensity distribution and the number distribution. Abbreviations: CHX = cyclohexane, NP-5 = Igepal CO-520, NH3 = ammonia solution (28 wt% in water), MeOH 1 = 50 μL methanol (121 mM) and MeOH 2 = 150 μL (359 mM) | | | | | | | | |
| --- | --- | --- | --- | --- | --- | --- | --- | --- |
| Sample | d_Int_ (nm) Peak 1 | ±_Int_ (nm) Peak 1 | d_Int_ (nm) Peak 2 | ±_Int_ (nm) Peak 2 | d_No_ (nm) Peak 1 | ±_No_ (nm) Peak 1 | d_No_ (nm) Peak 2 | ±_No_ (nm) Peak 2 |
| CHX + NP-5 | 3.5 | 1.3 | 0 | 0 | 1.9 | 0.5 | 0 | 0 |
| CHX + NP-5 + NH3 | 6.3 | 2.2 | 0 | 0 | 3.6 | 1.0 | 0 | 0 |
| CHX + NP-5 + NH3 + MeOH 1 | 7.8 | 4.0 | 1709.1 | 734.7 | 3.3 | 1.0 | 0 | 0 |
| CHX + NP-5 + NH3 + MeOH 2 | 7.9 | 3.7 | 3852.9 | 986.6 | 3.7 | 1.0 | 0 | 0 |

*Quantum yield calculations with the nanocavity model*

The upper limit of the quantum yield (QY) of the silica coated LaPO_4_:Eu^3+^(5%) and LaPO_4_:Ce^3+^(1%),Tb^3+^(5%) was determined using the method described by Senden et al [3]. This method does not correct for the refractive index of silica around the NCs which affects the local field correction factor. Calculations using a method describing the local field correction factor for a core-shell system were based on Formula 3.1 and 3.3 in ref. [4]. The radiative decay rate of emitters in the NCs is given by

$$\Gamma_{r}\left( n \right)=\Gamma_{0}nB^{2} (eq. S1)$$

where Γ_0_ is the radiative decay rate in bulk material (0.31 ms^-1^ for LaPO_4_:Eu^3+^ and 0.29 ms^-1^ for LaPO_4_:Tb^3+^), *n* = 1.36 (ethanol) and B is the local field factor and is described by:

$$B=\frac{9n^{2}n_{shell}^{2}}{\left( n_{shell}^{2}+2n^{2} \right)\left( n_{NC}^{2}+2n_{shell}^{2} \right)+2\beta\left( n_{NC}^{2}-n_{shell}^{2} \right)\left( n_{shell}^{2}-n^{2} \right)} (eq. S2)$$

where *n*_NC_ = 1.79, β= (d_LaPO4_/d_SiO2_)^3^ and *n*_shell_ is the refractive index of the silica shell surrounding the LaPO_4_ nanocrystal (NC). The refractive index of amorphous silica is approximately 1.45. However, the refractive index of the shell is influenced by the solvent in the pores of the silica. For this reason, *n*_shell_ was varied from the refractive index of the solvent (1.36) to the refractive index of amorphous silica (1.45). In this way, a minimum and a maximum value for B was obtained, where the minimum value represents the situation where no shell around the NC is present. Next, two radiative decay rates with QY = 1 were calculated. Figure S2 shows the measured decay curves and the two calculated decay curves for the europium doped LaPO_4_ NCs coated with silica (left) and for the terbium doped LaPO_4_ NCs coated with silica (right). The QY of the emitters coated with silica was calculated by dividing the area under the measured decay curve by the area under the theoretically determined decay curves. The calculated QY for the europium doped silica particles is 0.45 and 0.46 for calculations with the lowest and highest local field fill factors, respectively. QY values of 0.55 and 0.57 are obtained for the terbium doped silica particles.

| 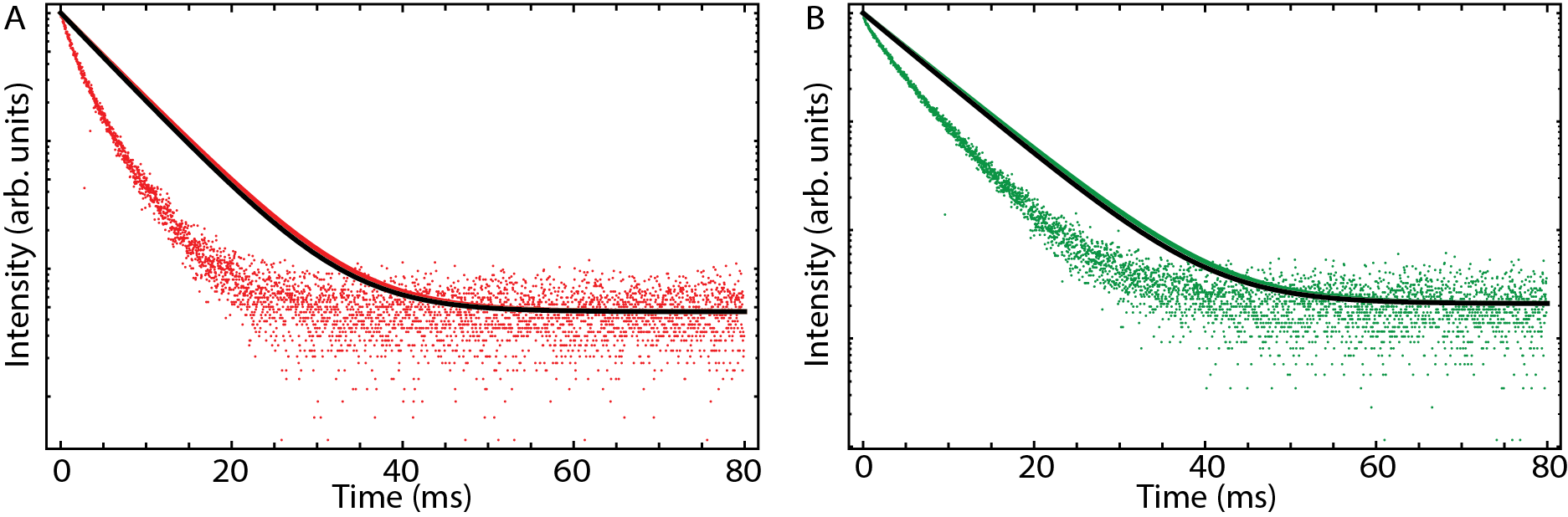 |
| --- |
| Figure S2. A: Decay curve of 611 nm Eu^3+^ emission for 4 nm LaPO_4_:Eu^3+^ NCs incorporated in 28 nm silica spheres dispersed in ethanol excited at 465 nm, the radiative decay curve with QY = 1 calculated with the lowest local field factor obtained in eq. S2 (red line), τ = 6.5 ms and QY = 0.45 and the radiative decay curve with QY = 1 calculated with the highest local field factor obtained in eq. S2 (black line), τ = 6.2 ms and QY = 0.46. B: Decay curve of LaPO_4_: Ce^3+,^Tb^3+^ NCs incorporated in silica spheres dispersed in ethanol excited at 487 nm, the radiative decay curve with QY = 1 calculated with the lowest local field factor obtained in eq. S2 (green line), τ = 6.9 ms and QY =0.55 and the radiative decay curve with QY = 1 calculated with the highest local field factor obtained in eq. S2 (black line), τ = 6.6 ms and QY = 0.57. |

**References**

[1]: K. Hickmann, K. Kömpe, A. Hepp and M. Haase, *Small*, 2008, **4**, 2136-2139

[2]: R. Koole, M. M. van Schooneveld, J. Hilhorst, C. de Mello Donega, D. C. ’t Hart, A. van Blaaderen, D. Vanmaekelbergh and A. Meijerink, *Chemistry of Materials*, 2008, **20**, 2503-2512

[3]: T. Senden, F. T. Rabouw and A. Meijerink, *ACS Nano*, 2015, **9**, 1801-1808

[4]: P. Lavallard, M. Rosenbauer and T. Gacoin, *Physical Review A*, 1996, **54**, 5450-5453
